# Giant Mesozoic coelacanths (Osteichthyes, Actinistia) reveal high body size disparity decoupled from taxic diversity

**DOI:** 10.1038/s41598-021-90962-5

**Published:** 2021-06-03

**Authors:** Lionel Cavin, André Piuz, Christophe Ferrante, Guillaume Guinot

**Affiliations:** 1 Department of Geology and Palaeontology, Natural History Museum of Geneva, Geneva, Switzerland; 2 Department of Earth Sciences, University of Geneva, Rue des Maraîchais 13, 1205 Geneva, Switzerland; 3 Institut des Sciences de L’Evolution de Montpellier (Université de Montpellier, CNRS, IRD, EPHE), Montpellier, France

**Keywords:** Evolutionary theory, Palaeontology

## Abstract

The positive correlation between speciation rates and morphological evolution expressed by body size is a macroevolutionary trait of vertebrates. Although taxic diversification and morphological evolution are slow in coelacanths, their fossil record indicates that large and small species coexisted, which calls into question the link between morphological and body size disparities. Here, we describe and reassess fossils of giant coelacanths. Two genera reached up to 5 m long, placing them among the ten largest bony fish that ever lived. The disparity in body size adjusted to taxic diversity is much greater in coelacanths than in ray-finned fishes. Previous studies have shown that rates of speciation and rates of morphological evolution are overall low in this group, and our results indicate that these parameters are decoupled from the disparity in body size in coelacanths. Genomic and physiological characteristics of the extant *Latimeria* may reflect how the extinct relatives grew to such a large size. These characteristics highlight new evolutionary traits specific to these “living fossils”.

## Introduction

Body size is often used as a proxy for analyzing morphological disparity, and this element is one of the main evolutionary traits discussed by biologists and paleontologists in order to decipher macroevolutionary processes. For example, a general increase in body size over time within animal lineages was one of the earliest nomological law in biology raised by Cope and Depéret^1^, and has subsequently been regularly confirmed for various clades^2^. Recent studies have shown positive correlations between speciation rates and morphological evolution expressed in body size^3,4^. However, body size is only one of the many traits that characterize morphological disparity, which can be measured by many other parameters^5^, and the assumption that body size disparity directly reflects morphological disparity can be questioned.

Coelacanths form a depauperate group of sarcopterygian fish with only one genus today but with a long evolutionary history. These fish are nicknamed "living fossils" because they possess characteristics used by Darwin to characterize this ill-defined concept, in particular "new forms … have been more slowly formed"^6^ (Darwin, however, did not cite the coelacanths, known only from fossils at that time, as examples of "living fossils"). Indeed, the clade exhibits low taxic diversity since its origins in the Devonian (ca 420 Mya) with approximately 63 genera in total. Only three weak successive peaks of higher taxic diversity are recorded in the Upper Devonian, in the Early Carboniferous and in the Middle Triassic^7^. Huxley (1866)^8^ has already noticed the low anatomical disparity of coelacanths throughout their history, and this observation has been confirmed by most subsequent studies^9–15^. This monotonic rate of evolution is interrupted by at least three episodes of increased morphological disparity, with forms presenting a different Bauplan, roughly contemporary with peaks of taxic diversity^9,16,17^. The rate of genetic evolution within the coelacanth lineage is found by most studies to be slower than that of other vertebrate lineages in the mitochondrial genome^18–20^ as well as in the nuclear genome^21^, at least for the protein-coding genes^22,23^.

Extinct giant coelacanths, i.e. fish several meters long, have long been described among the mawsoniids with *Mawsonia gigas* by Woodward in 1907^24^, from the Early Cretaceous of Brazil, then with *Axelrodichthys lavocati* from the ‘mid’ Cretaceous of North Africa^25,26^, with *Trachymetopon* sp.^27^ from the Middle Jurassic of Europe, but also among the latimeriids with *Megalocoelacanthus* from the Late Cretaceous of North America^28,29^. The fossil record of coelacanths reveals a relative abundance of large-sized species as previously observed by Wenz^25^ and Dutel et al.^27,30^.

Here we describe new fossil remains from the Middle Jurassic of Normandy, France, representing one of the largest known coelacanths ever reported. The specimen, a piece of a braincase, was found in the same deposits as fragmentary fossils interpreted as possible pups of the same species. We further reassess the Mesozoic fossil record of giant coelacanths and provide a large-scale comparison of body size disparity versus taxic diversity between coelacanths (Actinistia) and ray-finned fishes (Actinopterygii) over the Devonian–Paleocene time interval. We show that the per genus coefficient of body size variance is higher in coelacanths than in ray-finned fishes. This result calls into question the positive correlation between speciation rates and body size found in most vertebrate lineages, and more generally questions the use of body size as a valid proxy for anatomical disparity^3,4^.

## Results

### New material, geographical and stratigraphic provenances

**Figure 1:**
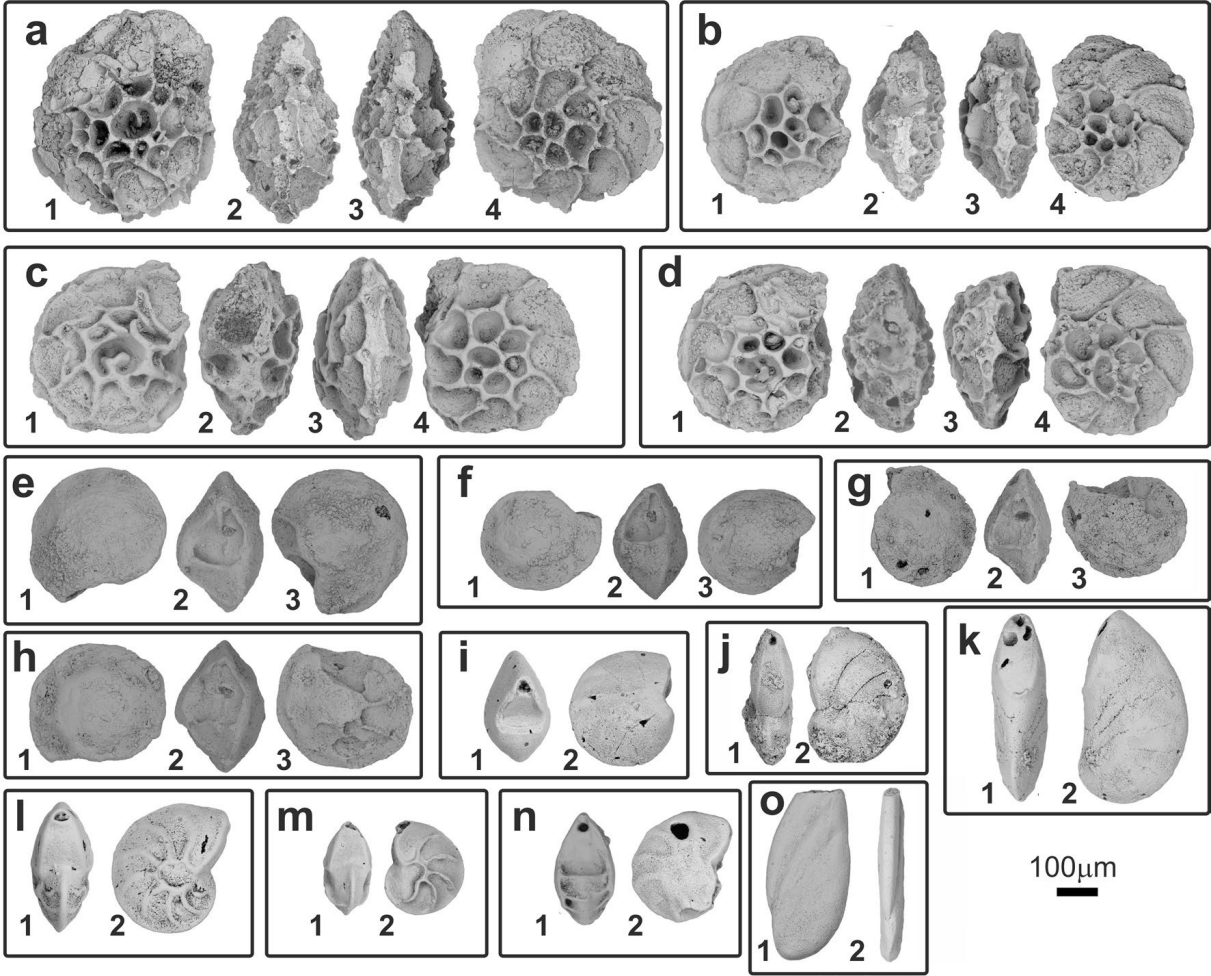
Foraminifera found in the matrix containing the fragment of the coelacanth skull (MHNG GEPI V5778). (**a**–**d**) *Epistomina* ex. gr. *mosquensis* Uhlig 1883, umbilical, apertural, carinal and spiral views; (**e**–**h**) *Epistomina* ex. gr. *uhligi* Mjatliuk 1953, spiral, apertural and umbilical views; (**i**–**k**) *Lenticulina muensteri* (Roemer 1839), apertural and lateral views; (**l**–**m**) *Lenticulina quenstedti* (Gümbel 1862), apertural and lateral views; (**n**) *Lenticulina subalata* (Reuss 1854), apertural and lateral views; (**o**) *Planularia beierana* (Gümbel 1862), lateral and apertural views.

**Figure 2:**
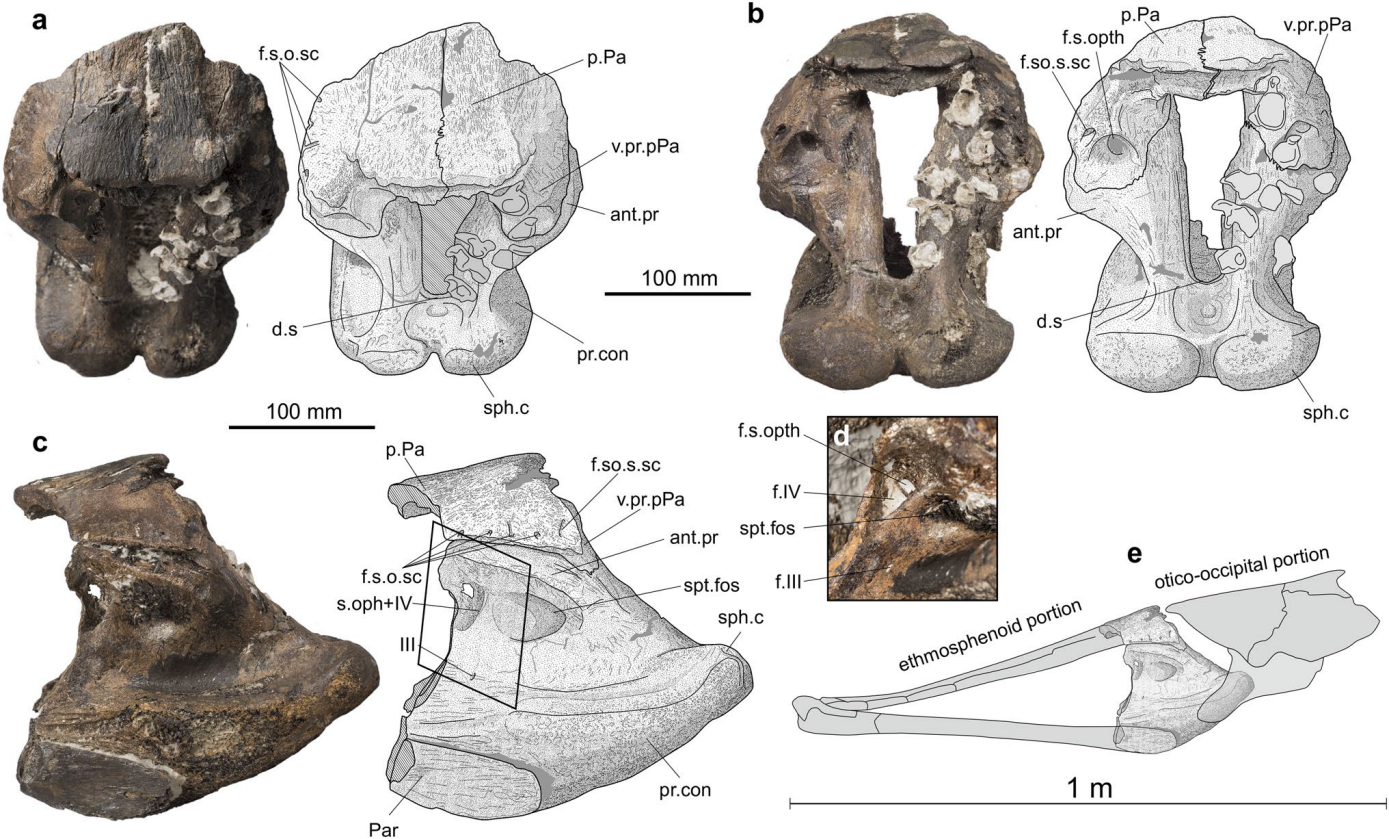
MHNG GEPI V5778. *Trachymetopon* sp. Basisphenoid with fragments of the posterior parietals and parasphenoid. Dorsal (**a**), dorsoposterior (**b**) and left lateral views (**c**). **d**, detail of exits of the nerve in anterolaterodorsal view (corresponding approximately to the frame in **c**); **e**, position of the fossil in a schematic reconstruction of the braincase of a mawsoniid coelacanth (modified from Maisey, 1986). Abbreviations: d.s, dorsum sellae; f.s.o.sc, foramen for the supraorbital sensory canal; f.s.opth, foramen for the superficial ophthalmic nerve; s.oph + IV, opening for the superficial ophthalmic nerve and the trochlear nerve; III, foramen for the oculomotor nerve; f.IV, foramen for the trochlear nerve (IV); ant. pr, antotic process; Par, parasphenoid; pr.con, processus conectens; p.Pa, posterior parietal; sph.c, sphenoid condyle; spt.fos, suprapterygoid fossa; v.pr.pPa, ventral process of the parietal posterior.

A large, almost complete, basisphenoid of a coelacanth with the posterior end of the parasphenoid sutured ventrally and the posterior part of the posterior parietals sutured dorsally (MHNG GEPI 5778) has been spotted in the paleontological collections of the Geneva Natural History Museum, Switzerland. No labels nor information were associated with this specimen. A search in the museum’s archives to trace the origin of the specimen was unsuccessful. The fossil was mechanically and chemically prepared, with 10% diluted HCl. The sediment recovered during the preparation of specimen was prepared by acetolysis in order to extract microfossils. The material recovered includes vertebrae and teeth of small fish, diverse micro-gastropods, as well as micro-bivalves, crinoids (roveacrinids), bryozoans, and foraminifera. The diversity of foraminifera is relatively low, consisting of moderately preserved epistominids and vaginulinids. The recognized species (Fig. 1) are *Epistomina* ex. gr. *mosquensis* Uhlig 1883, *Epistomina* ex. gr. *uhligi* Mjatliuk 1953, *Lenticulina quenstedti* (Gümbel 1862), *L. muensteri* (Roemer 1839), *L. subalata* (Reuss 1854) and *Planularia beierana* (Gümbel 1862). These taxa have only moderate biostratigraphic value, being mainly widespread in the upper Middle to Upper Jurassic (Supplementary Fig. S1). The presence of a modern barnacle shell on the fossil (Supplementary Fig. S2A), an evidence of its discovery from a locality situated near the seashore, associated to the general type of preservation of the specimen and to the presence of vaginulinids and epistominids associated with vertebrate fossils are strong indications that this specimen probably comes from the late Callovian “Marnes de Dives”, probably from the well-exposed cliffs of the “Vaches Noires”, Villers-sur-Mer, Normandy, France^31^. This facies generally contains an abundance of encrusted gryphaeid oysters as seen on the skull of the coelacanth bearing several of these shells on one of its sides (Supplementary Video, Fig. 2, Supplementary Fig. S2B). In addition to these shells, the matrix of the specimen bore the imprint of an ammonite, reminiscent of *Heticoceras* (Christian Meister, Antoine Pictet, personal communication 2014) and a gastropod shell (Supplementary Fig. S2C, D). The “Marnes de Dives” are the equivalent of the lower part of the “Oxford Clay” of Dorset, UK. It is assumed that the specimen reached the Geneva Natural History Museum near its inception in 1820 (it was then called the Academic Museum), along with other fossil vertebrates from Normandy, France.

Recently, two small basisphenoids of coelacanths (MPV 2020.1.13) were discovered by Elisabeth and Gérard Pénnetier from a Callovian strata of the foreshore at the foot of the cliffs of the “Vaches Noires”, Villers-sur-Mer, Normandy, France, and therefore come from the same formation as the large basisphenoid.

### Morphological description and comparisons

The large specimen (MHNG GEPI V5778) consists of a complete basisphenoid, in connection dorsally with the posterior part of the skull roof and ventrally with a fragment of the parasphenoid (Fig. 2). The processus connectens are well developed, slightly curved in lateral view and extend ventrally to the level of the parasphenoid. The dorsum sellae is proportionally short and forms anteriorly a shallow wall that constricts ventrally the entrance to the cranial cavity. On the posterior side of the bone, the well-developed and closely spaced sphenoid condyles are separated from each other by a marked notch and from the paired processus connectens by shallow depressions. The opening of the cranial cavity is much deeper than wide, with its dorsal part slightly wider than its ventral part. The antotic processes protrude laterally and suture dorsally to the ventral processes of the posterior parietal. The surfaces of contact between both processes are large and oval. The suprapterygoid fossa is well marked but shallow. Anteriorly, a large foramen opens oriented frontwards. Within the ossification, just behind the opening, the canal is divided by a thin horizontal lamina, which separates a larger ventral canal from a smaller dorsal one (Fig. 2d). Based on the paths of the nerves in *Latimeria*^11^, the small dorsal canal may have accommodated the superficial ophthalmic nerve and the ventral canal the trochlear nerve (IV). In *Latimeria*, but also in most post-Paleozoic taxa in which this part of the braincase is preserved, such as *Megalocoelacanthus*^29^ and *Trachymetopon*^30^, the nerves exit the cranial cavity in the interorbital cartilage. In the Devonian genera *Diplocercides* and *Euporosteus*^11^, the nerves exit through bone-enclosed foramens. We interpret the occurrence of this large foramen in MHNG GEPI V5778 as a consequence of the large size of the individual and its high degree of ossification rather than affinities with Paleozoic forms. Ventral to the large foramen, and present on both sides of the basisphenoid, opens the small oculomotor foramen. The angle between the posteroventral surface of the basisphenoid and the ventral surface of the parasphenoid is approximately 135°. The basipterygoid and suprapterygoid processes are absent.

The posterior parts of both posterior parietals are still sutured to the basisphenoid. The portion exposed to the surface of the skull roof, ornamented with faint anastomosed longitudinal ridges, is divided in two parts: a horizontal median part and an inclined lateral part on each side, forming an angle of approximately 120° with the horizontal part in posterior view. At the posterolateral edge of the inclined part of the postparietal opens a large foramen for the entry of supraorbital sensory canal. Along the lateral margin of the left preserved part of the posterior parietal open four small pores for the supraorbital sensory canal. The descending process of the posterior parietal extends ventroposteriorly from the inclined part of the bone. Posteriorly opens a large rounded foramen for the superficial ophthalmic nerve.

Although no diagnostic characters of *Trachymetopon liassicum* identified by Dutel et al.^30^ are observable on the specimen, except its large size, several features allow referring this material to this species: in lateral view the basisphenoid is triangular in shape with a curved lateral margin and a short dorsum sellae; the antotic process and processus connectens are well developed, the latter reaching the parasphenoid; the opening for the cranial cavity is deeper than wide and its outline is quadrangular (slightly wider dorsally than ventrally in our specimen); a marked notch separates the short and divergent sphenoid condyles; and the angle between the posteroventral surface of the basisphenoid and the ventral surface of the parasphenoid is approximately 135°. The only significant difference between the material of *T. liassicum* described by Dutel et al.^30^ and ours is that the anterior margin of the intracranial joint is straight in the former, while it has two marked notches in the latter. We notice, however, that the material of *T. liassicum* from Holzmaden figured by Hennig^32^ and Dutel et al.^30^ is apparently not very well preserved in this area. Consequently, MHNG GEPI V5778 is referred here to *Trachymetopon* sp. The general morphology of the specimen is also very similar to those of other mawsoniids, in particular *Mawsonia* and *Axelrodichthys*. Estimation of the total length of this individual based on proportions of the type specimen of *T. liassicus* is 5 m (see below).

### Evidence of potential pups of *Trachymetopon* sp

**Figure 3:**
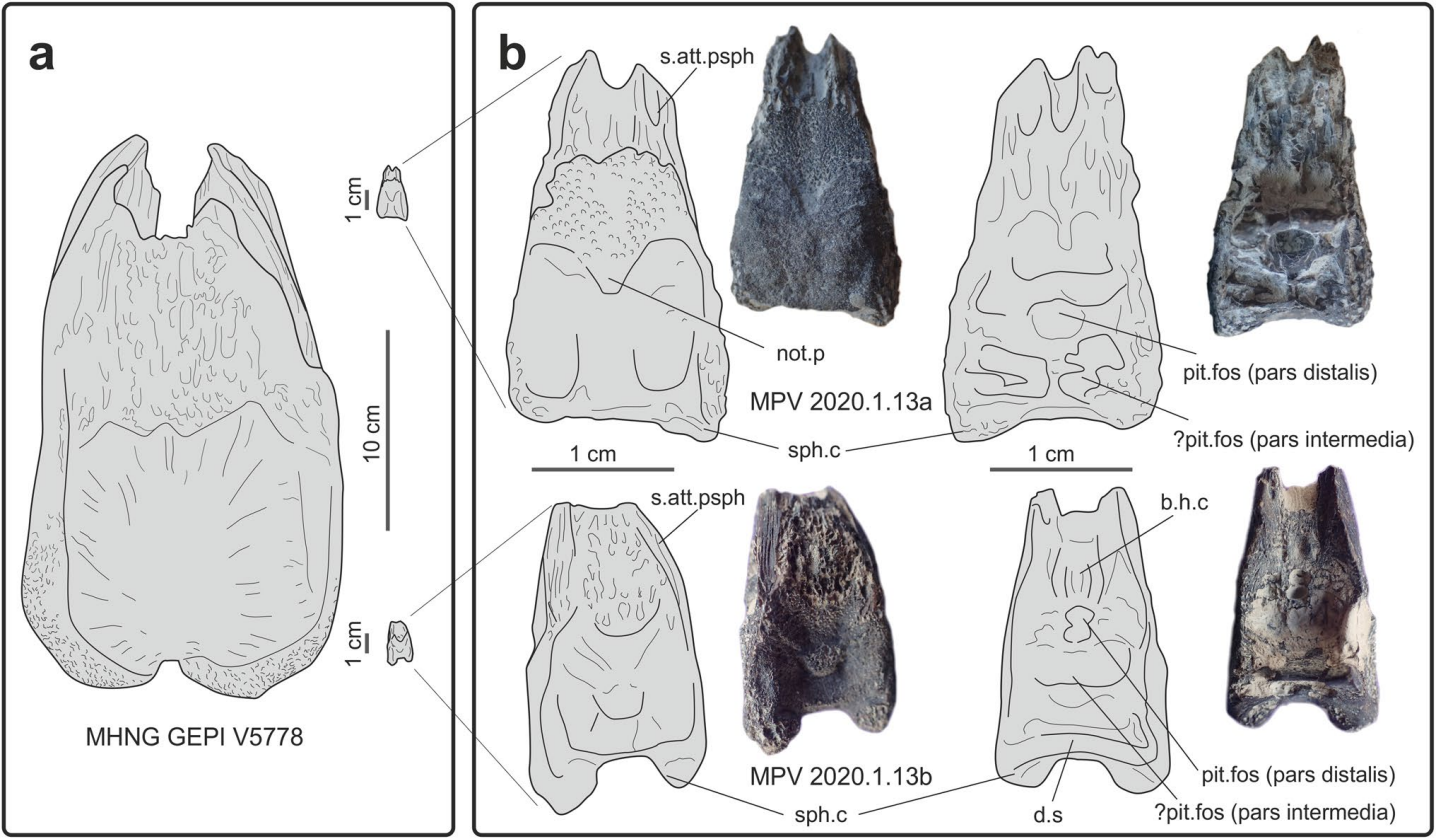
Basisphenoids of embryos or newborns of *Trachymetopon* sp. from Callovian beds from the Vaches Noires. (**a**) Comparison of the giant specimen (MHNG GEPI V5778) with basisphenoids of potential pups (MPV 2020.1.13); (**b**) details of the basisphenoids of the potential pups (MPV 2020.1.13a and MPV 2020.1.13b) in ventral (left) and dorsal (right) views. Abbreviations: b.h.c, buccohypophysal canal; d.s, dorsum sellae; not.p, notochordal pit; pit.fos, pituitary fossa; s.att.psph, surface of attachment for the parasphenoid; sph.c, sphenoid condyle.

Two small basisphenoids of coelacanth (Fig. 3 and Supplementary Fig. S3; MPV 2020.1.13a & b) were found by Elisabeth and Gérard Pennetier in the Callovian beds from the Vaches Noires. These specimens were donated to the Paléospace Muséum, France.

Both specimens consist of ventral part of the basisphenoids only, i.e. the paired processus connectens connected via a bony surface against which abutted the notochord, the paired sphenoid condyles and the dorsum sellae in one specimen (Fig. 3b). In the largest specimen (MPV 00.1.13a), the sphenoid condyles are widely separated and not very protruding, probably for preservational reasons. The slightly smaller specimen (MPV 00.1.13b) is better preserved than the larger one. The internal side of the both specimens shows well-marked reliefs. On the anterior part of the bone, the ventral side and the lateral sides bear strong grooves for suturing with the parasphenoid.

Although both specimens are very incomplete, we tentatively refer them to *Trachymetopon* sp. because a marked notch separates the short and divergent sphenoid condyles and the angle between the posteroventral surface of the basisphenoid and the ventral surface of the parasphenoid is approximately 135° (slightly more in MPV 2020.1.13.a). Specimen MPV 2020.1.13.a retained the base of the pila antotica, which marks with the horizontal line an angle similar to that of the large specimen (approximately 150°, Supplementary Fig. S3). In ventral view, the length-to-width ratios are roughly similar for large and small specimens. This ratio is equivalent to nearly 1.8 in our material, while it is significantly lower in most other coelacanths.

If this identification is confirmed, both small specimens obviously belong to two young individuals by comparisons of their size with MHNG GEPI V5778. In MPV 2020.1.13a, a shallow notochordal pit is still visible ventrally between the two processus connectens (Figs. 3 and Supplementary Fig. S3). In the fetus of *Latimeria*, the tip of the notochord passes through the basisphenoid in the notochordal foramen, which closes during development and leaves a notochord pit present in young individuals. Moreover, while the inner surface of the basisphenoid is generally smooth in adult individuals of various species of coelacanths, with only a pituitary notch between the antotic processes and overhung by the dorsum sellae (e.g. *Moenkopia wellesi*^33^; *Macropoma lewesiensis*^11^), it is carved by strong cavities and ridges in the two small specimens described here. The fossae probably indicate the presence of a compact pituitary gland by comparison with the development of brain in fetus and pups of *Latimeria*^34^.

Hypophyseal development in *Trachymetopon*: In the adult *Latimeria*, the brain occupies about 1% of the space of the cranial cavity, and the ventral floor of the basisphenoid is dug by the pituitary fossa (pit. fos), which accommodates the enlarged anterior portion of pars distalis (adenohypophysis) of the pituitary gland, while the posterior part of the gland, including the bipartite pars intermedia is located much more posteriorly beneath the optic chiasm in the otico-occipital portion of the braincase^11,35^. Dutel et al.^34^ showed that the pituitary gland underwent strong modifications during ontogeny in *Latimeria*. At fetal stage, the brain is proportionally very large and occupies both ethmosphenoid and otico-occipital cavities, and the pituitary gland is compact and lies under the diencephalon. During growth, the gland rotates dorsally towards the telencephalon when the brain is being concentrated in the posterior part of the cavity, while the anterior extremity of the pars distalis remains at the level of the basisphenoid and connects to the pars intermedia by the hypophyseal duct. The buccohypophysal canal (b.h.c) closes early during ontogeny^36^. Based on the observation in *Latimeria*, we consider that both small basisphenoids belong to pups of *Trachymetopon*, and we tentatively interpret the reliefs on their floor as following: a posterior bilobate fossa possibly accommodated the bipartite pars intermedia of the pituitary gland, while a median deep cavity situated just anteriorly corresponds to the pituitary fossa of adult coelacanths, i.e. it accommodated the anterior part of the pars distalis. The latter corresponds to the adenohypophysis, which secretes among other growth hormone, and the well-developed bony cavity indicate that the gland was proportionally larger than in corresponding stages in *Latimeria* (^34^, Extended Data Figs. 3b,c and 4b,c). Both partes are still close to each other because of the early stage of development. This arrangement of the hypophysis would correspond to the stages pup 1 or pup 2 of Dutel et al.^34^ based on their Extended Data Fig. 4. A groove anterior to the pituitary fossa accommodated the remnant of the buccohypophyseal canal. It is not possible to figure out if the hypophyseal fossa was still open because of preservation.

**Figure 4:**
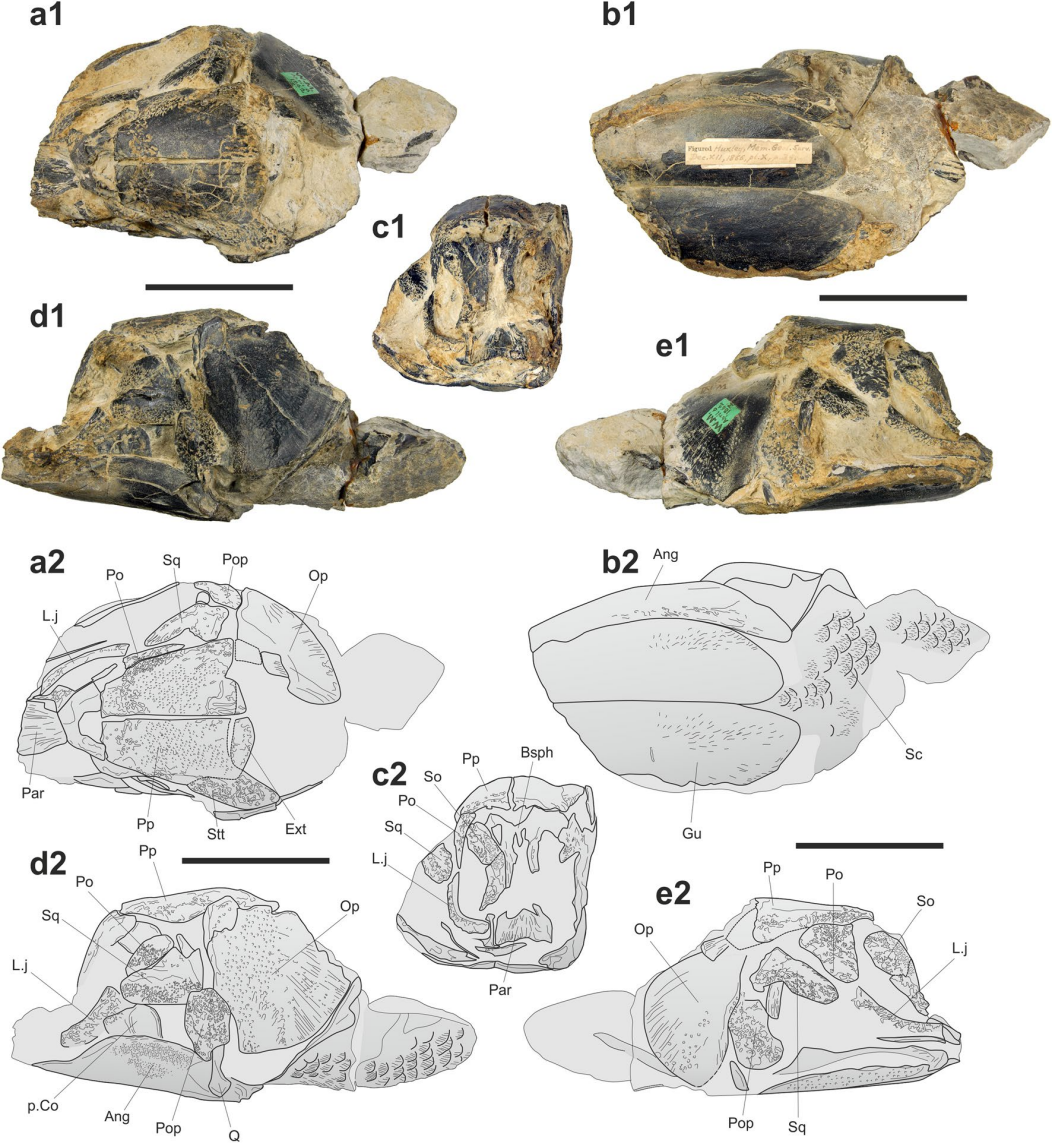
*Trachymetopon* (“*Macropoma*”) *substriolatum* (holotype, SMC J27415) from the Kimmeridgian of Cottenham, Cambridgeshire. Photograph (**1**) and semi-interpretative drawings (**2**) in dorsal (**a**), ventral (**b**), anterior (**c**), left lateral (**d**) and right lateral (**e**) views. Scale bars = 50 mm. Abbreviations: Ang, angular; Bsph, basisphenoid; Ext, extrascapular; Gu, gular; L.j, lachrymojugal; Op, opercle; Par, parasphenoid; p.Co, principal coronoid; Pop, preopercle; Pp, postparietal; Po, postorbital; Q, quadrate; Sc, scale; So, supraorbital; Sq, squamosal; Stt, supratemporal.

### *Trachymetopon* stratigraphical range

So far, the genus *Trachymetopon* is known in the Early and Middle Jurassic of Europe, but new data require re-evaluation of this stratigraphic range. *Trachymetopon liassicus* from the Toarcian (Early Jurassic) of Holzmaden, Germany, was named and described by Hennig^32^ and revised by Dutel et al.^30^, who referred it to the family Mawsoniidae on the basis of a cladistic analysis. The holotype is a complete and articulated specimen of 1.6 m in length, i.e. in the length range of the extant *Latimeria*. *Trachymetopon* was then discovered in the Middle Jurassic with giant forms discovered in Callovian strata in Normandy, France, described by Dutel et al.^27^ and in the present study.

In addition to this material, the Etches collection Museum at Kimmeridge, Dorset, UK, houses skull elements of a coelacanth from the Kimmeridgian (K785) that reached about 1.5 m in length compared to skeletal proportions of the complete type specimen. It consists of an angular, a quadrate, a metapterygoid and partial pterygoid, of a paired ceratohyals, of a cleithrum and indeterminate bones. The shape and ornamentation of the angular, and the proportion of the palatoquadrate are reminiscent to mawsoniids, and more specifically *Trachymetopon*. It is referred here with caution to *Trachymetopon* sp.

The holotype and only known specimen of *“Macropoma*” *substriolatum* (SMC J27415; Fig. 4) from the Kimmeridgian of Cottenham, Cambridgeshire, UK, was originally included in the genus *Macropoma* by Huxley^8^, then *Coccoderma* by Reis^37^ and Woodward^38^, and eventually brought closer to *Holophagus* by Forey^11^. In this specimen, the supratemporals appear to be restricted to the posterior lateral margin of the postparietal shield, and extend posteriorly creating a space that was occupied by the extrascapulars (preserved as fragments), like in mawsoniids and some other coelacanth genera. The strong ornamentation of the skull roof with conspicuous ridges and grooves is another mawsoniid character. Similar to *Trachymetopon*, the quadrate is massive, broad and has a convex anterior margin, the angular is long and low with a straight outline. The cheek is composed of a lachrymojugal, a postorbital, a squamosal and a preopercle, which are all thick and proportionally large bones with coarse ornamentation, as in mawsoniids. A difference with *T. liassicum* is that some bones strongly ornamented in the latter species (e.g., the postparietal, the angular, the opercle) are almost smooth with only a dense pattern of small pits in *T. substriolatum*. However, these parts of the specimen are the most exposed ones and we suspect that they were worn possibly before fossilization or, more probably, once the fossil was exposed to the surface. The specimen can be referred to a mawsoniid, and we provisionally refer this species to *Trachymetopon*. Based on our model, this individual was small, about 60 cm in length.

Based on this short review, we consider with confidence that the stratigraphic range of *Trachymetopon*, previously restricted to the Early and Middle Jurassic, extends to the Late Jurassic.

### A review of giant Mesozoic coelacanths

**Figure 5:**
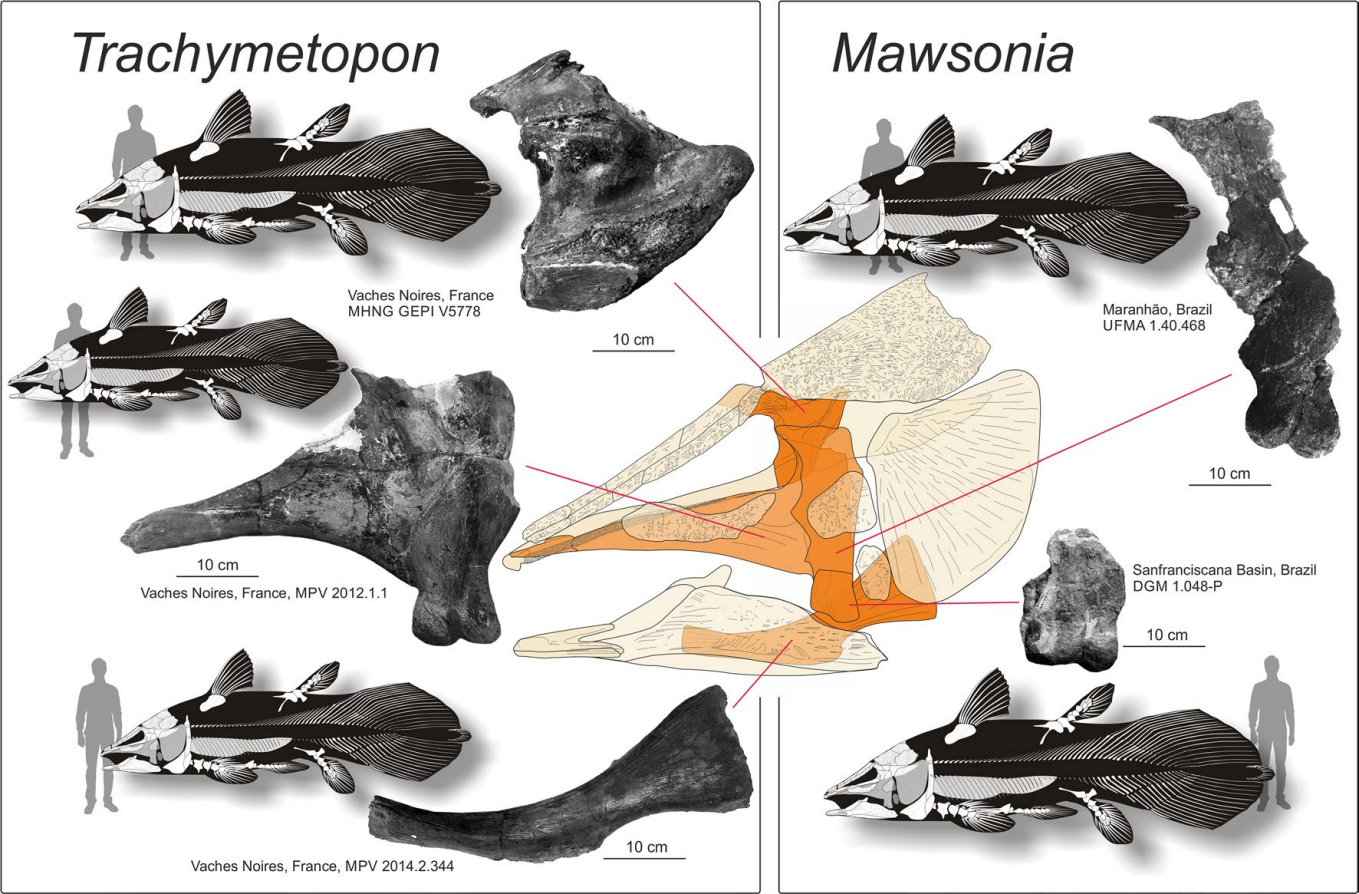
Fragmentary elements from the giant specimens of the Jurassic *Trachymetopon* and the Cretaceous *Mawsonia*. Human silhouettes corresponds to 1.8 m.

First remains of giant mawsoniids from the Early Cretaceous of Brazil were originally misinterpreted as belonging to a giant pterosaur by Woodward^39^ because of the peculiar biconvex articular condyle of the quadrate. This author then recognized his error with more complete material from the Recôncavo Basin, Brazil, that he named *Mawsonia gigas* based on its obvious large body size^40^. Based on our model (see Material & Methods), the body length of the holotype individual reached 3.1 m in length. *Mawsonia* bones were later found in various Early Cretaceous South American basins mostly Brazil but also Uruguay^41,42^, mainly represented by fragmentary elements corresponding to middle-sized individuals, but also to giant ones. One specimen coming from the Neocomian of Bahia is an articular head of a quadrate (DGM 1.048-P) and corresponds to an individual of 6.3 m in length according to Carvalho & Maisey^43^. Examination of this specimen by one of us (LC) and estimation based on our model indicates a total body length of 5.3 m (Fig. 5). Medeiros et al.^44^ recorded from the Laje do Coringa flagstone, Alcântara Formation in northeastern Brazil, a fragment of a large pterygopalatine comprising the quadrate, the metapterygoid and a piece of the pterygoid (UFMA 1.40.468). Based on our model, this specimen was 4.9 m long. African Cretaceous mawsoniids also reached meters-long sizes^25,26^, but never as long as *Trachymetopon* or as South American mawsoniids. The sister genus of *Mawsonia* is *Axelrodichthys*, which lived for part of its time interval in sympatry with *Mawsonia* in Brazil^45^, and extends to the Late Cretaceous in Europe with smaller forms^46^. In Africa *'Mawsonia' lavocati* has been referred to *Axelrodichthys* by Fragoso et al.^47^, and remains of this species from the Kem Kem Group in Morocco indicate individuals up to 3.5 m long^48^. Recently, Brito et al.^49^ referred an ossified lung fragment from the Late Maastrichtian of Morocco to an undetermined mawsoniid. Besides being the last record of a fossil coelacanth, the individual was a giant with an estimated total body size of between 3.65 m and 5.52m^49^. The fossil was recovered from marine sediments, but it is still uncertain whether the fish lived in this environment or whether the corpse was transported from a river system, as was the case with continental animals found in the same deposits^50–52^.

Dutel et al.^27^ referred an isolated palatoquadrate (MPV 2012.1.1) found in the Callovian (Middle Jurassic) of the Vaches Noires, France, to *Trachymetopon* sp. This specimen corresponds to a large individual estimated to reach 4 m in length. In addition to this large pterygoid, we mention here a large ceratohyal (MPV 2014.2.344) found in the same Callovian beds of the Vaches Noires, housed in the Paléospace Museum (Fig. 5). We estimate that this bone corresponds to an individual slightly larger than the one represented by the pterygoid, i.e. 4.4 m in length.

Among the latimeriids, *Megalocoelacanthus dobiei* is a giant species from the Late Cretaceous of North America known by disarticulated and mainly cranial elements. Several estimates of body size have been proposed, i.e. between 3.8 and 4.0 m for the holotype specimen (CCK 88–2-1) calculated by Schwimmer^28^ and between 2.3 and 3 m for another specimen (AMNH FF 20,267) calculated by Dutel et al.^29^. Based on the basisphenoid of the holotype and comparison with to the body proportions of *Latimeria*, we obtained a total length of 3.5 m for the latter specimen.

The body length estimates for the largest known specimens are summarized in Table 1.

**Table 1:** Calculated body length (B.L., in meters) for some of the largest specimens of *Trachymetopon* spp., *Mawsonia gigas*, *Axelrodichthys lavocati* and *Megalocoelacanthus dobei*.

*Trachymetopon* spp.		*Mawsonia gigas*		*Axelrodichthys lavocati*		*Megalocoelacanthus dobei*	
Specimens	B.L	Specimens	B.L	Specimen	B.L	Specimen	B.L
MHNG GEPI V5778	5.0	DGM 1.048-P	5.3	UMI-1	3.5	CCK 88–2-1	3.5
MPV 2014.2.344	4.4	UFMA 1.40.468	4.9				
MPV 2012.1.1	4.0						

### Body size evolution and disparity in coelacanths

Linear regression analysis between coelacanth logtransformed body length and time expressed in time bins spanning the Devonian-Cretaceous interval shows a statistically significant positive correlation (r = 0.42963; *p* < 0.0001), indicating a general trend for body size increase over time in Actinistia. The evolution of body size disparity in coelacanths is decoupled from the observed trends in taxic diversity (Pearson's product-moment correlation: 0.3450382; p-value = 0.2078).

**Figure 6:**
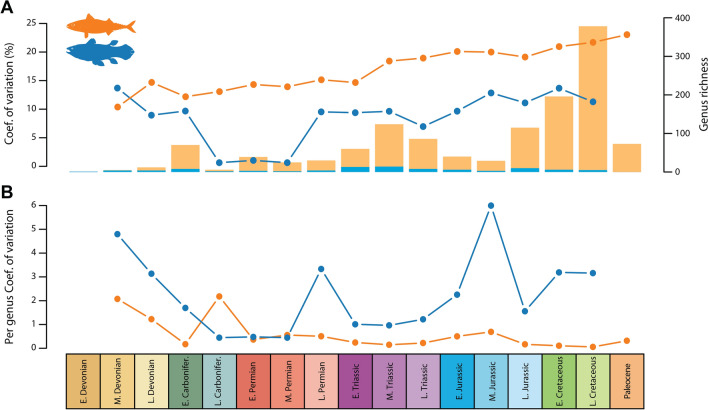
(**A**) range-through genus richness (histograms) and disparity expressed by the coefficient of variation (dots and lines); (**B**) per genus coefficient of variation, which is the coefficient of variation standardized by taxic diversity. Orange, Actinopterygii; blue, Actinistia.

We computed the coefficient of variation to quantify coelacanth body size disparity across 17 time bins spanning the Devonian-Paleocene interval, and used actinopterygians for comparison. Actinopterygians were chosen because their time range is comparable to that of coelacanths, and because of their enormous taxic diversity, which makes them representatives of the changes in body length disparity through time in about half of the vertebrate diversity. Our results (Fig. 6A) indicate that body size disparity is globally lower in coelacanths, with the exception of the earliest stages of their evolutionary history. However, the ray-finned fish taxic diversity is tremendously higher than that of coelacanths over the vast majority of their evolutionary history (Fig. 6A), which tends to make direct comparison of disparity misleading. Once standardized by taxic diversity, the per genus body size disparity patterns differ drastically (Fig. 6B). Coelacanths display a much higher per genus disparity than actinopterygians by several orders of magnitude throughout most of their evolutionary history, excepted in the late Carboniferous where actinopterygians show a high per genus disparity and in the Permian, where both clades present roughly similar disparity values.

## Discussion

The new fragmentary remains of *Trachymetopon* described here, and the body size reassessment of Jurassic and Cretaceous mawsoniids indicate the presence of individuals reaching or exceeding 5 m in total body length during the Jurassic and Cretaceous. By comparison, among the actinopterygians living from the Devonian to the Paleocene, the only genera which approach or exceed *Mawsonia* and *Trachymetopon* in length are two giant marine planktivorous pachycormiforms, the Jurassic *Leedsichthys* (16 m) and the Cretaceous *Bonnerichthys* (6.1 m), as well as the Late Cretaceous to Recent *Acipenser* (5 m). Among the extant ray-finned fish, the only longest species include another chondrostean, *Huso huso* (7.2 m) and the Atlantic blue marlin, *Makaira nigricans* (5 m), as well as the oarfish *Regalecus glesne* (13.7 m) but the latter has a compressed and slender profile very different from the other fish compared, all more or less fusiform shaped. Thus, the two genera of mawsoniid coelacanths are among the ten largest bony fish that have ever lived. Interestingly, one of these giant coelacanths, *Trachymetopon*, lived in sympatry in the European Callovian Sea with the largest ray-finned fish that ever lived, *Leedsichthys*^46^.

Fifteen genera of coelacanths are known in the Jurassic and Cretaceous, i.e. contemporaneous genera of the giant *Trachymetopon* and *Mawsonia*. They were medium-sized fish, but proportionately small compared to the two giants, with seven genera whose body length did not exceed 0.5 m *(Reidus, Swenzia, Macropomoides, Coccoderma, Atacamaia, Undina,* and *Lualabaea*). The smallest known coelacanth genera lived mainly in the Paleozoic (*Holopterygius, Lochmocercus, Hadronector,* and *Youngichthys*), then in the Triassic (*Piveteauia* and *Chaohuichthys*). The general mean increase in body size in this lineage is demonstrated by the correlation between body size and time, which confirms the Cope's rule previously observed in many clades^2^. Such a trend is not observed in actinopterygians as a whole, but is present in most of the main clades taken separately^54^, probably because testing the Cope’s rule gives contrasting results depending on the taxonomic level used^55^. Because of the proportionally smaller body size of the older coelacanths, and because small size might be considered as what Gould^56^ called the “left wall” in evolution of complexity, evolution of body size would predominantly lead to a passive trend towards larger body size (the “Stanley effect”^57^ according to Albert & Johnson^58^). Nevertheless, observation of the distribution of body size over time indicates that the lowest body size tends to increase from the Early Devonian to the Late Cretaceous (Supplementary Fig. S4), although this trend remains to be demonstrated statistically.

From this aspect, the coelacanths do not deviate from this general macroevolutionary trend observed in a majority of clades of actinopterygians^54^.

Our analyses of the evolution of body size disparity in coelacanths indicate high disparity from the Late Permian onward compared with actinopterygians. Although this pattern is not visible based on raw disparity values (Fig. 6A), it is clearly noticeable when disparity is standardized by taxic diversity, which allows to fit the much higher finned-ray diversity to the depleted coelacanth clade (Fig. 6B). A decrease is visible for both clades in the Late Jurassic, possibly caused by the Lagerstätten effect detected in the fossil record of ray-finned fish at this time^59^ which alters the measure of disparity by preserving a greater diversity and more complete specimens^60^. We also note that the variance in body size of ray-finned fish steadily increases over time when the index is taken raw, but when adjusted for diversity, the variance in body size is very stable during the Permian-Paleocene interval, possibly indicating that the body size ecospace was fulfilled during this time interval.

Previous studies have demonstrated that the coelacanth morphological disparity, whether measured by morphospace occupation^61^ or by computation of new discrete characters in a phylogenetic framework^9–12,14^, shows a burst at the origin of the group in the Devonian – Carboniferous. These studies confirmed the early burst (EB) model first proposed by Simpson^62^, which was then verified at a large scale among animal clades^63–65^, and demonstrated more specifically in the ichthyosaurs^66^. The same trend is observed here for body size disparity in the early evolutionary history of the coelacanths. However, the coelacanth pattern differs from that of other clades in that their body size disparity did not decrease through time, contrary to what is observed in the morphological evolution of ichthyosaurs for instance^66^. Instead, the evolution of the coelacanth body size disparity tended to increase until the late Cretaceous, then this evolutionary trend remains unknown due to the lack of fossils in the Cenozoic. Interestingly, our analyses indicate that the evolution of body size disparity in coelacanths is decoupled from the observed trends in taxic diversity. Such a decoupling between morphological disparity and taxic diversity after the initial radiation of a clade has been reported on many instances for various groups based on fossil evidence^67,68^, indicating that taxic diversity and disparity may be controlled by different factors (but see^3,4^). However, the post-Carboniferous variations in body size disparity through time among coelacanths does not mirror those of morphological evolution for this clade either^9–11^. Those studies on morphological evolution indicate a steady drop from the Carboniferous until the Cretaceous with some exceptions such as the aberrant *Foreyia* from the middle Triassic^17^, which is in sharp contrast with the pattern of body size disparity presented in Fig. 6. This indicates that body size might not be an accurate proxy for reflecting morphological evolution and/or disparity.

Underwater observations in situ^69,70^ and gill surface measurements^71–73^ of *Latimeria* all indicate a very low metabolic rate in this fish. Based on Kleiber's law stating that large animals have a proportionately lower metabolic rate than small ones, and although we cannot say whether a low metabolic rate is a cause or consequence of a large body size, the low rate observed in *Latimeria* can be considered as a trait inherited from the common ancestor of the latimeroids (mawsoniids plus latimeriids), which is associated in an indeterminate way to the gigantism of *Mawsonia* and *Trachymetopon*, and to the large size of *Axelrodichthys* and *Megalocoelacanthus*. The recent study^74^ of the genome of the giant whale shark (*Rhincodon typus*) and a comparison of genomic and physiological features of a set of 83 animals revealed several correlations between these life traits. In particular, these authors detected a negative correlation between length of introns in the genome and metabolic rate, and a positive correlation between length of introns and body size. *Latimeria* has proportionally long intron length (^74^, Fig. 1e, Supplementary Fig. S3), making its clade prone to evolve toward large body size.

Finally, the small basisphenoids provisionally attributed to pups of the giant *Trachymetopon* show that adenohypohysis was probably proportionally large in these young individuals. Edinger^75^ showed that the adenohypophysis is proportionally much larger in large animals, such as giant dinosaurs, thus confirming an older observation by Nopsca^76^, and in large birds and mammals. Although the available evidence is still weak, the proportionally large adenohypophysis in the young of *Trachymetopon* sp. may be associated with the large size of adults.

New fossil discoveries and an examination of the body size of coelacanths through time confirm that the evolutionary history of these fish is in agreement with two major macroevolutionary trends widely observed in animal evolution, namely an early burst in their morphological disparity (previously demonstrated) and a gradual increase in body size through (Cope/Depéret's rule), but they also deviate from two other macro-evolutionary trends, that is, their variations in body size disparity are not linked with taxic diversity nor with morphological evolution. The genomic characteristics, the long intron length and the physiological characteristics, the low metabolic rate of the extant *Latimeria* constitute a favorable ground for the evolution towards gigantism in this clade.

## Material and methods

### Microplalaeontological preparation

Microfossils have been extracted from a very small amount of rock residue retrieved from the preparation of the coelacanth bone. Due to the strong induration of the sediment, extraction of microfossils, unsuccessful with traditional washing methods^77^, have then been done by acetolysis^78^.

### Body size reconstruction

The model used to reconstruct the body length in Fig. 5 is based on the reconstruction of *Axelrodichthys araripensis* by Forey (^11^, Fig. 11.3), itself based on the reconstruction of Maisey^45^ with some additions. As far as the material allows to assess, there are no major differences in body proportions between *Axelrodichthys*, *Trachymetopon* and *Mawsonia*. Note that our model is based on individuals much smaller than the individuals studied here, and it is possible that allometric growth may alter the calculation of body size estimates. However, McAllister and Smith^79^ showed that in *Latimeria chalumnae* the length of the head, from the snout to the posterior end of the operculum, grows isometrically compared to the standard length. If, anyway, allometric growth was present in mawsoniids, it means that our body size reconstructions are underestimated because the allometry in fish, as in most vertebrates, involved a proportionately larger head in young individuals than in older and larger ones. All analyses were performed using R version 3.6.0^80^.

### Stratigraphical ranges and body size dataset

We gathered data on the fossil record (first and last occurrences) of each of the 63 coelacanth genera from the Devonian to the Paleocene. We further compiled the maximum body length for each genus based on the literature and/or on direct measurements of complete specimens or based on estimates for partial specimens. For species known by isolated skulls only, we multiplied the skull length (snout to posterior margin of the opercle) by 4.14, a ratio calculated on the basis of a sample of complete specimens of various species. For comparison purpose, we also gathered data for the actinopterygians. These are based on an update of Guinot & Cavin^54,81^, with new information from Sallan & Coates^82^ for Devonian taxa, Romano et al.^83^ for Permo-Triassic taxa and Alberts et al.^58^, complemented by extensive literature review. We used total length for both actinistian and actinopterygian genera. When only standard lengths were available for ray-finned fishes, we multiplied them by 1.2 for getting the total length, a ratio calculated on the basis of a large sample of taxa known by complete specimens. When different sizes were available for one species, we selected the longest one. When several species are known for a genus, we selected the longest one. Data are available in Supplementary Table S1.

### Stratigraphical ranges and body size analyses

We used the coefficient of variation to quantify body size disparity across time bins throughout the Devonian-Paleocene interval. The coefficient of variation is expressed here in percent, as follows:

1 $$C\nu=\sigma/\mu*100$$

where *σ* is the standard deviation and *µ* is the mean of the body size values. We computed the coefficient of variation for each of the 17 time bins, which represent geological Epochs. Because disparity values can be influenced by taxic diversity, we further divided values of *Cv* in each time bin by the corresponding value of taxic diversity (computed by range-through), thus providing a per genus coefficient of variation standardized by taxic diversity. This allowed comparisons to be made between disparity values of clades that differ drastically in taxic diversity, such as actinistians and actinopterygians. Body length values were log-transformed prior to the analyses.

## Supplementary Information

Supplementary Information 1.

Supplementary Information 2.

Supplementary Video 1.

## Abbreviations

DGM: Divisão de Geológia e Mineralogia, Departamento Nacional da Produção, Mineral, Rio de Janeiro, Brazil
CCK: Columbus (Georgia) College, USA
MHNG GEPI: Natural history Museum of Geneva, Switzerland (palaeontological collection)
MPV: Paléospace, palaeontological museum of Villers-sur-Mer, France
SMC: Sedgwick Museum, Oxford, UK
UFMA: Coleção Paleontológica da Universidade Federal do Maranhão, Brazil
UMI: University Moulay Ismail of Meknès, Morocco
