# Supplementary material for: Giant Mesozoic coelacanths (Osteichthyes, Actinistia) reveal high body size disparity decoupled from taxic diversity
Source: Sci Rep. 2021 Jun 3;11:11812. doi: 10.1038/s41598-021-90962-5 (PMC8175595; doi:10.1038/s41598-021-90962-5)
Supplement: Supplementary file 2 — Supplementary Information 2. [file 41598_2021_90962_MOESM2_ESM.pdf]

Giant Mesozoic coelacanths (Osteichthyes, Actinistia) reveal high body size disparity decoupled from taxic diversity

Lionel Cavin[1*], André Piuz[1], Christophe Ferrante[1] & Guillaume Guinot[2]

[1] Department of Geology and Palaeontology, Natural History Museum of Geneva, Geneva, Switzerland

[2] Institut des Sciences de l'Evolution de Montpellier (Université de Montpellier, CNRS, IRD, EPHE), Montpellier, France

* Corresponding author

Email: lionel.cavin@ville-ge.ch (LC)

Appendix 1

Microfaunal assemblage

Recovered material includes small fish vertebra and teeth, diverse micro-gastropods, together with micro-bivalves, roveacrinids, bryozoans, and foraminifers. Regarding the latter, the diversity is poor although probably due to the fact that the agglutinated and porcelaneous shells were most likely dissolved during acetolysis. The small amount of rock and the fact that only small fragments were available did not allow making thin sections to check the diversity and the presence/absence of shells eventually dissolved by acetolysis.

Foraminifer's diversity is quite poor, consisting of moderately preserved epistominids (mainly smooth forms, rare ornamented forms) and vaginulinids. All of them only have a moderate biostratigraphical value (Serie/Epoch level). Many shells are pyritised. Recognized species are *Epistomina* ex. gr. *mosquensis* Uhlig 1883*, Epistomina* ex. gr. *uhligi* Mjatliuk 1953*, Lenticulina quenstedti* (Gümbel 1862)*, L. muensteri* (Roemer 1839)*, L. subalata* (Reuss 1854)*, Planularia beierana* (Gümbel 1862).

Epistominids

Species of the benthic foraminifer *Epistomina* have been used extensively for biostratigraphy (e.g. Ohm 1967, Magniez-Jannin 1975, Hart, 1984, Ascoli 1976, Ascoli 1984, Wiliamson & Stam 1988) as their abundance is often high in Jurassic/Cretaceous assemblages. To the stage level, they are often used as biostratigraphic markers, but as stated by several authors (e.g. Wiliamson & Stam 1988), many of the species described are probable synonyms, and questions remains if some long ranging species are the same species or if they can they be separated as different taxa.

Two species of Epistominids have been identified in our material: *Epistomina* ex gr. *mosquensis* Uhlig 1883 and *Epistomina* ex gr. *uhligi* Mjatliuk 1953.

Epistomina ex gr. *mosquensis* Uhlig 1883

Fig. 1a-d

Our specimens are biconvex, low trochospiral, with umbilical side slightly more convex than the spiral side. 7-8 chambers in the last whorl. Size is between 380µm and 530µm. Ornamentation is high and evidence deep "cells" (reticulate pattern) always present on both spiral and umbilical faces. On the spiral face, the ornamentation (walls of the cells) follows the contour of the chambers of the two last whorls. On the umbilical face, the ornamentation show deep "cells" often (not systematically) showing a central collar (e.g. Fig. 1c, d) surrounded by numerous deep "cells". When the collar is not evidenced, the "cells" cover the first whorl(s). Sutures are radial on umbilical face, arched on spiral face. Pits (small "cells") may appear at the extremities of the sutures, at the contact with the central ornamentation as well as on the keeled periphery of the test, rarely "along" the suture on the umbilical face (e.g. Fig. 1d). Periphery with variable keel (due to preservation?) most often double, slightly oblique and with pits. Apertures are not clearly visible due to the poor preservation of the tests.

Remarks: *E*. ex gr. *mosquensis* are rare and are the only ornamented epistominids found in our sample. Regarding the high variability known in the literature for *E. mosquensis* (see e.g. Ascoli, 1984, Barnard et al. 1981, Cordey 196, Espitalie & Sigal, 1963, Gradstein 1978, Henderson 1997, Ohm, 1967, Pazdro 1969, Stam 1986), we propose to attribute our specimens to *Epistomina* ex gr. *mosquensis*. Indeed, as stated by Wiliamson & Stam 1988, many species have been described but a revision of many of them would be necessary.

Wiliamson & Stam 1988 propose the umbilical collar to be characteristic of the species and propose to place the specimens lacking this feature (and having instead many small "cells") in *E. regularis* (see discussion in Wiliamson & Stam 1988). Our specimens (Fig.1b) which do not (clearly) show the collar are however proposed to fall within the variability of *E. mosquensis* as the umbilical cells present in our specimens are obviously (and on a regular basis in our 7 specimens), larger than the "pitting" of *E. regularis,* and never cover the whole umbilical face as often observed in *E. regularis* (see e.g. *E. regularis* in Terquem 1883 fig. 1b, Ohm 1967 p.124 abb. 20, Pazdro 1969 pl. I fig. 1d, II fig.1y 1k, XIII fig.6, Stam 1986 pl. 3 fig.1, Smolen 2012 txt-fig.5, but not in Wiliamson and Stam 1988 pl. 4). Our specimens attributed to *E. ex* gr. *mosquensis* are close to those figured e.g. by Ohm pl. 17 fig. 7a,b, or Pazdro pl. 2 fig.3. but clearly differ (especially the umbilical face) from those e.g. of Stam 1986 pl. 1, Ascoli 1984 pl. 2.

Stratigraphy: The ages given by various authors for the different morphotypes attributed to *E. mosquensis* are summarized in Fig. S1. In Europe, this species is mainly known from the Callovian – Oxfordian while in Canada it mainly flourishes during the Kimmeridgian. The wide conception of this species makes its boundaries somewhat loose. Locally, it may show a more restricted repartition possibly due to paleoecological conditions.

Paleoecology: Stam (in Wiliamson & Stam 1988) proposed that *E. mosquensis* preferred relatively deep environments (> 250 m) with the shallower waters being dominated by nodosariid fauna.

***Epistomina* ex gr. *uhligi* Mjatliuk 1953**

Fig. 1e-h

Our specimens have a trochospiral and biconvex test with a smooth (not ornamented) test on both sides. Spiral side is slightly convex and umbilical side strongly convex with outstanding translucent umbilical boss. Size is 280µm to 450µm. Spiral side shows 7 to 9 chambers in the last whorl. Spiral sutures are almost radial and barely visible in SEM images due to their very low elevation. Rare specimens show "false or apparent" raised sutures due to the (better) dissolution of the chamber wall compared to the thicker and stronger sutural wall. Umbilical sutures are radial reaching the central massive umbilical boss of translucent shell material. Periphery is very sharp with keel. Aperture is oval areal and situated on the spiral side of the toothplate very remarkable when the last chamber is broken (e.g. Fig. 1e, f, h).

Remarks: The smooth Epistominids we attribute to *Epistomina* gr. *uhligi* are largely the dominant foraminifera in this sample. *E. uhligi* is considered with significant variability, and among the smooth middle to late jurassic epistominids, numerous authors suggest that many of the smooth species may be one and the same taxon (see e.g Ohm, 1967, Stam, 1986, Wiliamson & Stam 1988, Riegraph & Luterbacher 1989). Commonly cited *Epistomina parastelligera* (Hofker) (a species with considerable variations in its interpretation) then enters (together with other species, see ibid cum bilio) the morphological variation considered for *E. uhligi*. Our smooth specimens resemble those figured e.g. by Ascoli 1976, 1984 (same figure as Jansa et al. 1980), Stam 1986 and then enter the variability range proposed as *E.* ex gr. *uhligi* e.g. in terms of test size and convexity of the umbilical and spiral face. Following some authors (e.g. Shipp 1978 in the Oxford Clay) *E. parastelligera* often occurs in floods. This can be the case in our sample regarding the dominance of *E.* ex gr. *uhligi* in this sample. Following Hart et al. (2016), in the late Jurassic, epistominids often typify maximum flooding surface.

Stratigraphy: *E. uhligi* and its synonym *E. parastelligera* are known from the Bajocian to the Tithonian (see Fig. S1) but may be locally more restricted stratigraphically (possibly for

paleoecological reasons). As for *E. mosquensis*, the variability of the species concept makes it an only moderate stratigraphical marker. It seems to become extinct at the end of the Tithonian.

Vaginulinids

Few vaginulinids have been found in the sample. They are quite rare and most are small. They are attributed to *Lenticulina quenstedti* (Gümbel 1862), *Lenticulina muensteri* (Roemer 1839), *Lenticulina subalata* (Reuss 1854), and *Planularia beierana* (Gümbel 1862).

***Lenticulina muensteri* (Roemer 1839)**

Fig. 1i-k

Involute planispiral smooth shell, thick, biconvex, periphery rounded, periphery acute to sub-acute, sutures flush to very slightly raised. An umbilical boss may be present in the umbilical area.

Remarks: Most (of our few) specimens are rather small (300-480µm) and close coiled (Fig. 1i) although some shells do exhibit uncoiling (Fig.1j, k). Variation in coiling of *L. muensteri* is well illustrated in e.g. Cifelli (1960), Gordon (1966), or Morris (1982). Numerous morphotypes (with different species names) are evidenced by different authors (see e.g. Barnard 1960, Jendryka 1975, Morris, 1982, Bartenstein & Bolli 1986). This species is known to be very common throughout the oxford clay (Shipp, 1978; Barnard et al 1981).

Stratigraphic repartition: *L. muensteri* is a very common, worldwide distributed and conservative taxon, with a vast stratigraphic range, from the Late Sinemurian to Albian (Jendryka, 1975, Lutze 1960, Bartenstein & Bolli 1986; Zsiboras & Görög 2020 cum biblio).

Lenticulina quenstedti (Gümbel 1862)

Fig. 1l, m

Our specimens show the specific raised and arched sutures, the keel (distinct in the first chambers of the last whorl, usually absent in the last chambers), and a more or less well developed umbilical ring. Aperture is terminal, peripheral. 7 to 9 chambers are present in the last whorl. Shell size is small and diameter ranges between 230 and 360 μm.

Remarks: *L. quenstedti* (Gümbel) is considered a highly variable taxon often split in numerous different names (see e.g. Reigraf & Luterbacher 1989, Bartenstein 2000). It is rare in our material, although frequent in the Oxford Clay in England (e.g. Shipp 1978).

Stratigraphy: It shows a Toarcian to Tithonian range, but its probable synonymy with *L. ouachensis* (Sigal), may extend its repartition up to the Barremian or Lowermost Aptian (see Riegraf et al. 1984, Riegraf & Luterbacher 1989, Bartenstein 2000).

Lenticulina subalata (Reuss 1854)

Fig. 1n

Two specimens are attributed to this species which is closely coiled with sutures distinctly raised, periphery acute to subacute, and presence of an umbilical plug.

Remarks: *L. subalata* is very similar to *L. muensteri* (in which it intergrades, e.g. Barnard et al. 1981) but it is characterized by its sharp keel (at least in the first chambers), strongly raised sutural ribs, and an umbilical disc (or boss). Study of the (high) variation of *L. subalata* is proposed by Bhalla & Abas, (1975). *Lenticulina helios* (Terquem 1870) is considered as

junior synonym of *Lenticulina subalata* (Reuss 1854) following Dietze et al. 2017 and Zsiboras et Gorog 2020.

Stratigraphy: Known from the Pliensbachian to the Tithonian (see e.g. Jenkins & Murray 1989, Zsiboras & Görög 2020 cum biblio).

Planularia beierana (Guembel 186)

Fig. 1o

Smooth compressed test of 7 planispirally arranged chambers; sutures distinct, depressed. Last chamber broken.

Remarks: *P. beierana* can exhibit a wide variety of test shapes and ornamentations (e.g. Cordey 1962, Shipp 1978, Barnard et al. 1981). It is one of the most common species in the Oxford Clay (Barnard et al. 1981).

Concluding remarks: The microfauna retrieved from the sediment around the coelacanth cranial element does not allow very precise dating. The only well preserved foraminifera are rather long-ranging species mainly widespread during the upper Middle to Upper Jurassic. Compared with those known from Upper Jurassic Lagerstatten, we can suggest the piece of braincase may come from the Late Callovian “Marnes de Dives”, famous for its reference section East of Houlgate (Normandy, France), called “Les Vaches Noires”. It is well known for various vertebrate remains (fish, ichthyosaurs, crocodiles, sauropterygians and dinosaurs), in layers yielding (among others) nodosariids and epistominids (e.g. Rioult et al. 1989). The facies typically containing abundant gryphaea also reminds the one encrusting cranial element. These “Marnes de Dives” are equivalent of the lower part of the “Oxford clay” of Dorset.

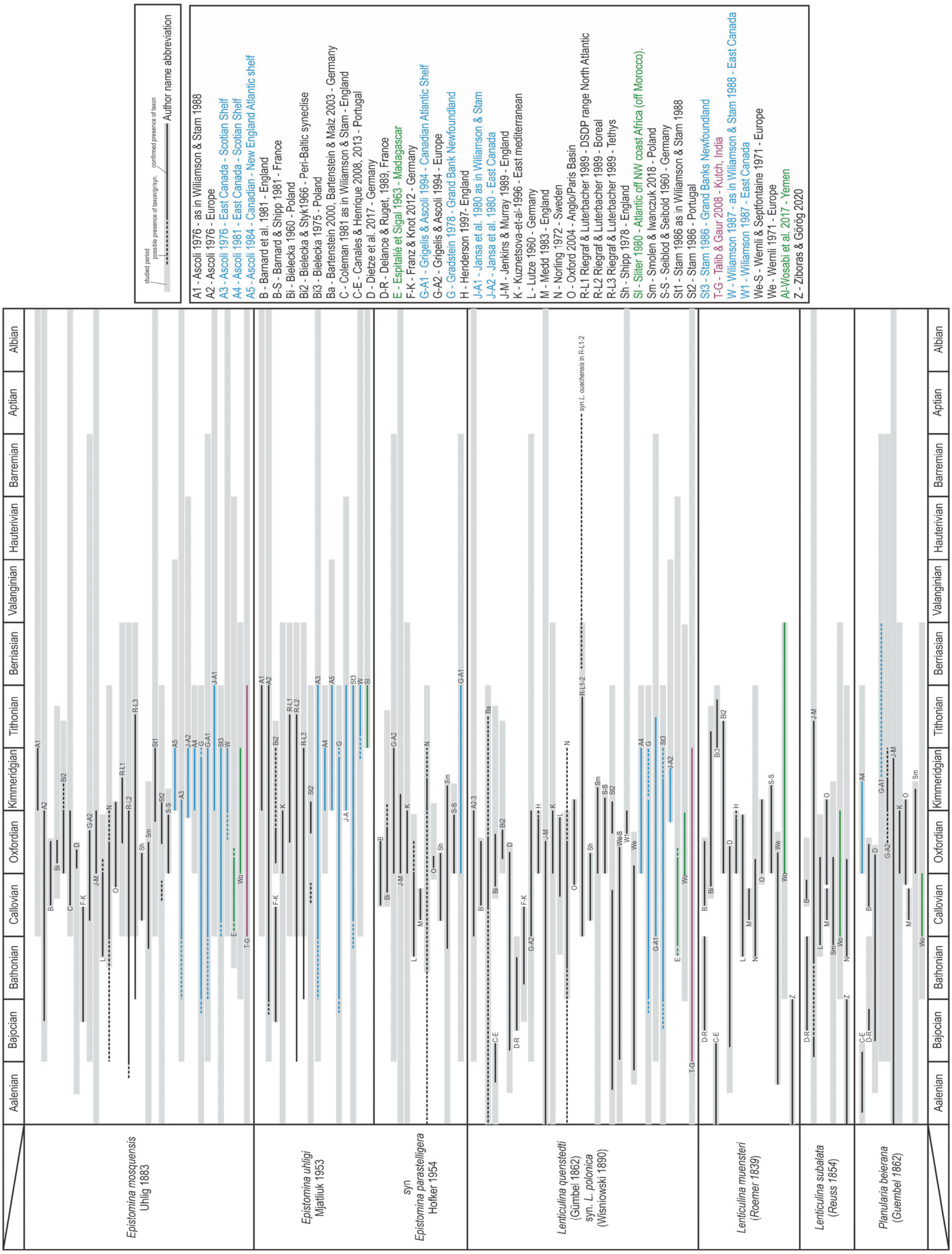

Figure S1:

Stratigraphic repartition of the foraminifers found associated with the piece of braincase following the literature.

References

Al-Wosabi, M., El-Anbaawy, M. & Al-Thour, K. Middle Jurassic–Early Cretaceous foraminiferal biozonation of the Amran Group, eastern Sana'a Basin, Yemen. *Geologos* **23**, 75-87 (2017).

Ascoli, P. Foraminiferal and ostracod biostratigraphy of the Mesozoic-Cenozoic, Scotian Shelf, Atlantic Canada. In: First International Symposium on Benthonic Foraminifera of Continental Margins, 1975. Proceedings, Part B., Maritime sediments, **S.P. 1.** Halifax, Nova Scotia, 653-771 (1976).

Ascoli, P. Foraminiferal-ostracod Late Jurassic biozonation of the Scotian shelf. Open file no. 753, Department of Energy, Mines and Resources, Ottawa, 1- 38 (1981).

Ascoli, P. *Epistomina* biostratigraphy across the Jurassic Cretaceous boundary on the northwestern Atlantic Shelf. Bu*ll. Centres recherches exploration-production Elf-Aquitaine* **6**, 27-34 (1984).

Barnard, T. Some species of *Lenticulina* and associated genera from the Lias of England. *Micropaleontology* **6**, 41-47 (1960).

Barnard, T., Cordey, W.G. &Shipp, D.J. Foraminifera from the Oxford Clay (Callovian–Oxfordian of England). *Revista Española de Micropaleontología* **13**, 383–462 (1981).

Barnard, T. & Shipp, D.J., Kimmeridgian Foraminifera from the Boulonnais. *Revue de Micropaléontologie* **24**, 3-26 (1981).

Bartenstein, H. *Lenticulina (Lenticulina) quenstedti* (Gümbel 1862) und ihre Variationsbreite im Mittel-und Oberjura Europas und Außereuropas. *Senckenbergiana lethaea* **80**, 405-425 (2000).

Bartenstein, H. & Malz, H. *Clarifovea* n.g. (Toarcium bis Tithonium) und *Grandifovea* n.g. (Valanginium bis Aptium), zwei neue mesozoische Foraminiferen-Gattungen mit 'ringförmiger' Nabelregion. *Senckenbergiana lethaea* **83**, 3-13 (2003).

Bartenstein, H. & Bolli, H.M. The foraminifera in the Lower Cretaceous of Trinidad, W.I. Part 5: Maridale Formation, upper Part; *Hedbergella rohri* Zone. *Eclogae Geologicae Helvetiae* **79**, 945-999 (1986).

Bartenstein, H. & Bolli, H. M. The Foraminifera in the Lower Cretaceous of Trinidad, WI Part 4: Cuche Formation, upper Part; *Leupoldina* protuberans Zone. *Eclogae Geologicae Helvetiae* **70**, 543-573 (1977).

Bhalla, S. N. & Abbas, S. M. A study of variation in *Lenticulina subalata* (Reuss). The *Journal of Foraminiferal Research* **5**, 145-148 (1975).

Bielecka, W. Stratygrafia mikropaleontologiczna osadów górnojurajskich Polski, bez Karpat. *Geological Quarterly* **4**, 949-963 (1960).

Bielecka, W. & Styk, O. Mikrofauna malmu południowej części syneklizy perybałtyckiej. *Geological Quarterly* **10**, 350-366 (1966).

Bielecka, W. Foraminifera and brackish Ostracoda from the Portlandian of Polish Lowlands. *Acta Palaeontologica Polonica* **20**, 295-407 (1975).

Canales, M. L. & Henriques, M. H. Foraminifera from the Aalenian and the Bajocian GSSP (Middle Jurassic) of Murtinheira section (Cabo Mondego, West Portugal): biostratigraphy and paleoenvironmental implications. *Marine Micropaleontology* **67**, 155-179 (2008).

Canales, M. L. & Henriques, M. H. Foraminiferal assemblages from the Bajocian global stratotype section and point (GSSP) at Cape Mondego (Portugal). *Journal of foraminiferal Research* **43**, 182-206 (2013).

Cifelli, R. Variation of English Bathonian Lagenidae and its phylogenetic significance. *Journal of Paleontology* **34**, 556-569 (1960).

Cordey, W. G. Foraminifera from the Oxford Clay of Staffin Bay, Isle of Skye, Scotland. *Senckenbergiana lethaea* **43**, 375-409 (1962).

Delance, J. H. & Ruget, C. Foraminifères du Dogger nivernais. *Revue de Micropaléontologie* **32**, 195-214 (1989).

Dietze, V., Franz, M., Kutz, M. & Waltschew, A. Stratigraphy of the Middle Jurassic Sengenthal Formation of Polsingen-Ursheim (Nördlinger Ries, Bavaria, Southern Germany). *Palaeodiversity* **10**, 49-95 (2017).

Epistalié, J. & Sigal, J. Contribution à l'étude des Foraminifères (Micropaléontologie-Microstratigraphie) du Jurassique supérieur et du Néocomien du Basin de Majunga (Madagascar). *Annales Géologique de Madagascar* **32**, 1-110 (1963).

Franz, M. & Knott, S. D. Foraminifera from the Callovian GSSP candidate section of AlbstadtPfeffingen (Middle Jurassic, Southern Germany). *Neues Jahrbuch für Geologie und Palaontologie Abhandlungen* **264**, 263-282 (2012).

Gordon, W. A. Variation and its significance in classification of some English Middle and Upper Jurassic Nodosariid foraminifera. *Micropaleontology* **12**, 325-332 (1966).

Gradstein, F. M. Jurassic grand banks foraminifera. *The Journal of Foraminiferal Research* **8**, 97-109 (1978).

Grigelis, A. & Ascoli, P. Middle Jurassic–Early Cretaceous Foraminiferal Zonation and Paleoecology of Offshore Eastern Canada and the East European Platform. *Geological Survey of Canada Atlantic. Open File* **3099**, 1-16 (1994).

Hart, M. The Superfamily Robertinacea in the Lower Cretaceous of the UK and adjacent areas of NW Europe. *Bull. des Centres de recherches exploration-production Elf-Aquitaine* **6**, 289-298 (1984).

Hart, M. B., De Jonghe, A., Page, K. N., Price, G. D. & Smart, C. W. Exceptional accumulations of statoliths in association with the Christian Malford lagerstätte (Callovian, Jurassic) in Wiltshire, United Kingdom. *Palaios* **31**, 203-220 (2016).

Henderson, A.S. *The palaeoecology and biostratigraphy of the foraminifera from the Oxfordian of North Dorset*. Ph.D. Thesis, University of Plymouth, 385p (1997).

Jansa L.F., Remane J. & Ascoli P. Calpionellid and foraminiferal-ostracod biostratigraphy at the Jurassic-Cretaceous boundary, offshore eastern Canada. *Rivista Italiana Paleontologia Stratigraphia* **86**, 67-126 (1980).

Jendryka-Fuglewicz, B. Evolution of the Jurassic and Cretaceous smooth-walled *Lenticulina* (Foraminiferida) of Poland. *Acta Palaeontologica Polonica* **20**, 99-197 (1975).

Jenkins, D.G., Murray, J.W. (Eds.) *Stratigraphical Atlas of Fossil Foraminifera*, 2nd ed. Ellis Horwood, Chichester. 593 p. (1989).

Kuznetsova, K.I., Grigelis, A.A., Adjamian, J., Jamarkini, E. & Hallaq, L. *Zonal Stratigraphy and Foraminifera of the Tethyan Jurassic (Eastern Mediterranean)*. Gordon et Breach Publishers, Amsterdam. 255p. (1996).

Lutze, G.F. Auf Stratigraphie und Paläontologie des Callovien und Oxfordien in Nordwest-Deutschland. *Geologisches Jahrbuch* **77**, 391–532 (1960).

Magniez-Jannin, F. Les Foraminifères de l'Albien de l'Aube: paléontologie, stratigraphie, écologie. *Cahiers de Paléontologie*. Éditions du C.N.R.S., Paris, 360 pp. (1975).

Medd, A. W. Foraminifera from the Lower Oxford Clay (Callovian Stage) of the Norman's Cross Pit, near Peterborough, Cambridgeshire. *Revista española de micropaleontologia* **15**, 221-240 (1983).

Morris, P. H. Distribution and palaeoecology of Middle Jurassic foraminifera from the Lower Inferior Oolite of the Cotswolds. *Palaeogeography, Palaeoclimatology, Palaeoecology* **37**, 319-347 (1982).

Norling, E. Jurassic stratigraphy and foraminifera of western Scania, southern Sweden. *Sveriges geologiska undersökning, Ser. C* **47**, 1-120 (1972).

Ohm, U. Zur Kenntnis der Gattungen *Rheinholdella, Garantella* und *Epistomina* (Foramin.). *Palaeontographica Abteilung A* **127**, 103-188 (1967).

Oxford, M. J. *Foraminiferal distribution and sequence stratigraphy of Oxfordian successions in the Wessex/Anglo-Paris Basin*. Department Geological Sciences University of Plymouth, 444p. (2004).

Pazdro, O. Middle Jurassic Epistominidae (Foraminifera) of Poland. *Studia Geologica Polonica* **27**, 1-92 (1969).

Riegraf, W. & Luterbacher, H. Oberjura-Foraminiferen aus dem Nord-und Südatlantik (Deep Sea Drilling Project Leg 1–79). *Geologische Rundschau* **78**, 999-1045 (1989).

Rioult, M., Coutard, J.-P., de La Quérière, P., Helluin, M., Larsonneur, C. & Pellerin, J. *Notice explicative de la feuille de Caen au 1/50000.* Editions du BRGM, 1-104. (1989)

Shipp, D. J. *Foraminifera from the Oxford clay and corallian of England and the Kimmeridgian of the Boulonnais, France*. Doctoral dissertation, University College London (University of London), 377p. (1978).

Sliter, W. V. Mesozoic foraminifers and deep sea benthic environments from Deep Sea Drilling Project Sites 415 and 416, eastern North Atlantic. Initial Rep. *Deep Sea Drilling Project* **50**, 353-370 (1980).

Smoleń, J. Faunal dynamics of foraminiferal assemblages in the Bathonian (Middle Jurassic) ore-bearing clays at Gnaszyn, Kraków-Silesia Homocline, Poland. *Acta Geologica Polonica* **62**, 403-419 (2012).

Smoleń, J. & Iwańczuk, J. Foraminiferal biostratigraphy of the Middle and Upper Jurassic of the Polish Lowlands: the state of the art. *Geological Quarterly* **62**, 257-286 (2018).

Siebold, E. & Siebold, I. Foraminiferen der bank-und Schwamm-Fazies im unteren Malm Süddeutschlands. *Neues Jahrbuch für Geologie und Paläontologie, Abhandlungen* **109**, 309-438 (1960).

Stam, B. Quantitative analysis of middle and late Jurassic foraminifera from Portugal and its implications for the Grand Banks of Newfoundland. *Utrecht Micropaleontological Bulletins* **34**, 1-168 (1986).

Talib, A. & Gaur, K. N. Foraminiferal composition and age of the Chari Formation, Jumara Dome, Kutch. *Current science* **95**, 367-373 (2008).

Terquem, O. *Cinquième mémoire sur les foraminifères du système oolithique de la zone à Ammonites Parkinsoni de Fontoy (Moselle)*, Metz, France, p. 379. (1883).

Wernli, R. Les foraminifères du Dogger du Jura méridional (France). *Archives des Sciences* **24**, 305-364 (1971).

Williamson, M. A. A quantitative foraminiferal biozonation of the Late Jurassic and Early Cretaceous of the East Newfoundland Basin. *Micropaleontology* **33**, 37-65 (1987).

Williamson, M. A. & Stam, B. Jurassic/Cretaceous Epistominidae from Canada and Europe. *Micropaleontology* **34**, 136-158 (1988).

Zsiborás, G. & Görög, Á. Aalenian–lower Bajocian foraminiferal fauna from the Ammonitico Rosso series of Bakonycsernye (Hungary). Part 1: Taxonomy and biostratigraphy. *Journal of Foraminiferal Research* **50**, 41-72 (2020).

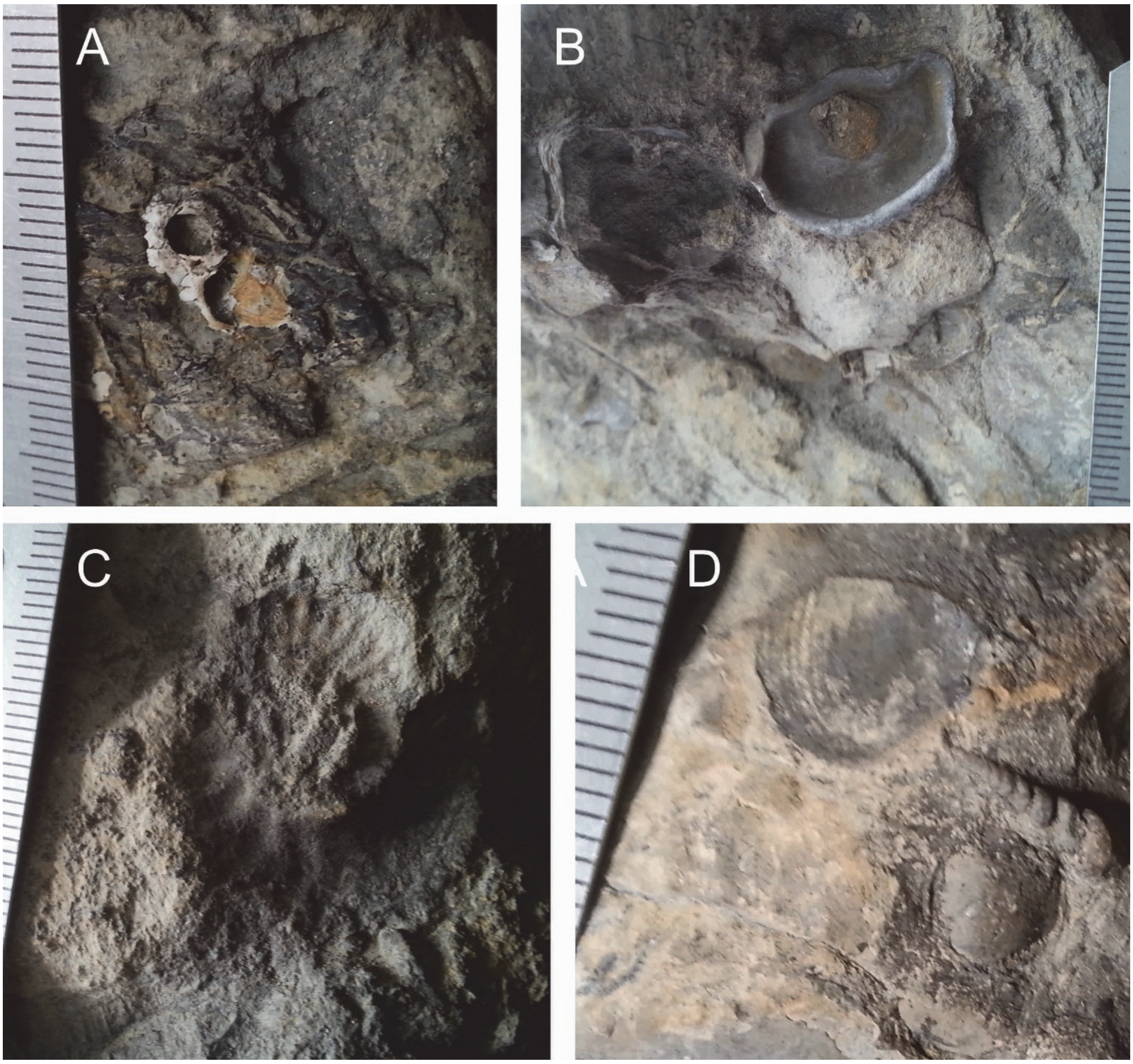

Figure S2:

Biological traces present on the matrix of the specimen before preparation.

A, a modern barnacle shell indicating that the fossiliferous locality was on the shore; B, a gryphaeid oyster attached on the coelacanth bone before fossilization; C, the imprint of an ammonite reminiscent of *Heticoceras*; D, shells an indeterminate gastropod and bivalve.

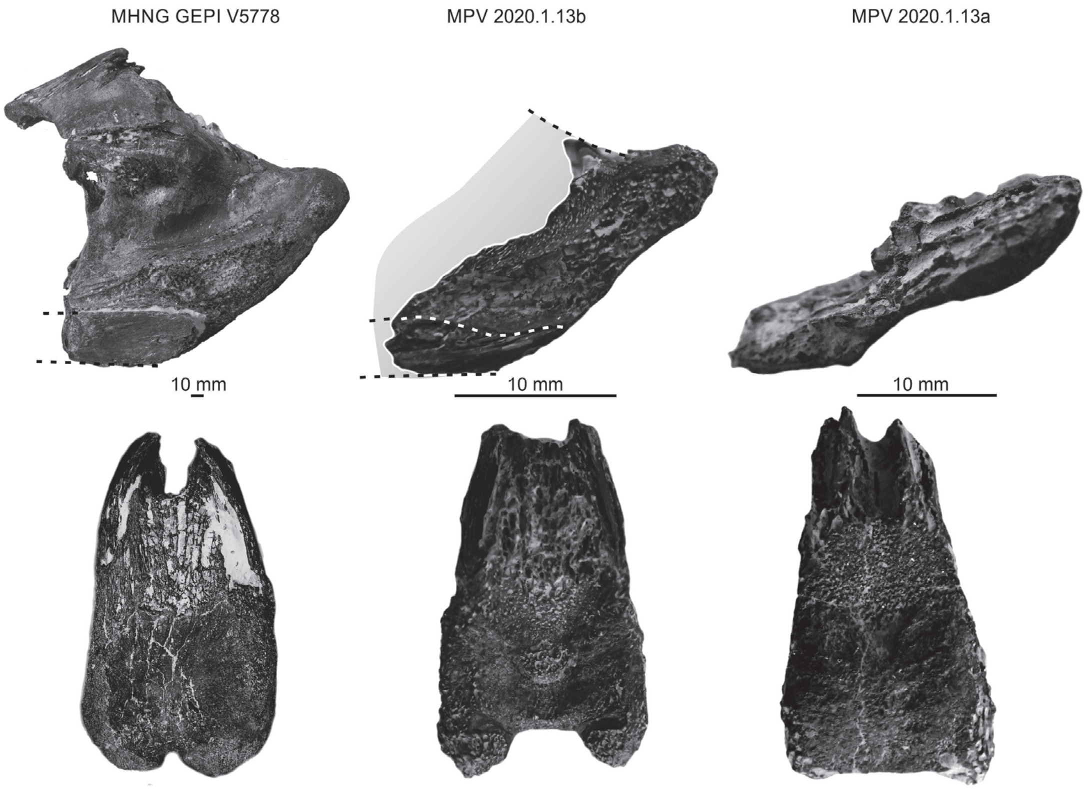

Figure S3:

Comparison of the adult (left) and pup (center and right) specimens of *Trachymetopon* sp. in lateral (up) and ventroposterior (down) views.

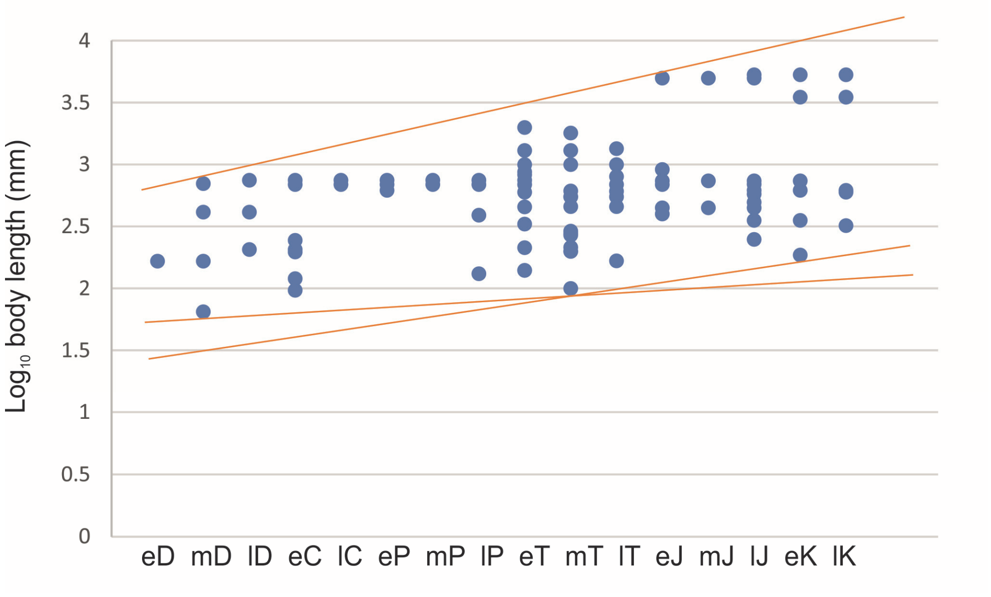

Figure S4:

Log of body length versus time for coelacanth genera

Abbreviations: C, Carboniferous; D, Devonian; e, Early; J, Jurassic, K, Cretaceous; l, Late; m, Middle; P, Permian; T, Triassic.

Table S1

Body size and stratigraphic ranges of genera of coelacanths (Actinistia) and ray-finned fish (Actinopterygii) from the Devonian to the Palaeocene.
